# Relationship between weight status and health-related quality of life in Chinese primary school children in Guangzhou: a cross-sectional study

**DOI:** 10.1186/s12955-016-0567-7

**Published:** 2016-12-03

**Authors:** Wei Liu, Rong Lin, Weijia Liu, Zhongshan Guo, Lihua Xiong, Bai Li, K. K. Cheng, Peymane Adab, Miranda Pallan

**Affiliations:** 1 Faculty of School Health, Guangzhou Centre for Disease Control and Prevention, Guangzhou, China; 2 Public Health, Epidemiology and Biostatistics, Institute of Applied Health Research, College of Medical and Dental Sciences, University of Birmingham, Edgbaston, Birmingham, B15 2TT UK

**Keywords:** Weight status, Children, Health-related quality of life

## Abstract

**Background:**

To investigate the association between weight status and health-related quality of life (HRQOL) among pupils in Guangzhou, China.

**Methods:**

The study comprised 5781 children aged 8–12 years from 29 schools. Height and weight were objectively measured using standardized methods, and BMI z-score derived using the age and sex specific WHO reference 2007 for 5–19 years. Weight status was classified as underweight (<−2SD), healthy weight (between -2SD and 1SD), overweight/obesity (>1SD). HRQOL was measured by the self-report version of the Pediatric Quality of Life Inventory 4.0.

**Results:**

After controlling for gender, age, school type, parental education, and family income, HRQOL scores were significantly lower in overweight/obese compared with healthy weight children only in the social functioning domain (*β* = −1.93, *p* = 0.001). Compared with healthy weight children, underweight children had significantly lower total (*β* = −1.47, *p* = 0.05) and physical summary scores (*β* = −2.18, *p* = 0.02). Subgroup analysis for gender indicated that compared to healthy weight, total (*β* = −1.96, *p* = 0.02), psychosocial (*β* = −2.40, *p* = 0.01), social functioning (*β* = −3.36, *p* = 0.001), and school functioning (*β* = −2.19, *p* = 0.03) scores were lower in overweight/obese girls, but not boys. On the other hand, being underweight was associated with lower physical functioning (*β* = −2.27, *p* = 0.047) in girls, and lower social functioning (*β* = −3.63, *p* = 0.01) in boys. The associations were mainly observed in children aged 10 and over, but were not significant in younger children. Children from private schools had generally lower HRQOL compared to those in public schools, but the associations with weight status were similar in both groups.

**Conclusions:**

The relationship between overweight/obesity and HRQOL in children in China is not as prominent as that seen in children in western or high-income countries. However, there appears to be gender and age differences, with more of an impact of overweight on HRQOL in girls and older children compared with boys and younger children. Underweight is also associated with lower HRQOL. Future intervention to prevent both obesity and undernutrition may have a positive impact on the HRQOL in children in China.

## Background

During recent years, the prevalence of overweight and obesity in children and adolescents has increased dramatically worldwide, especially in low and low middle-income countries [1, 2]. In China, a country which has been undergoing a substantial economic transition in the past few decades, the prevalence of childhood overweight and obesity has increased from 1.1 to 9.6% between 1985 and 2010 [3]. Although the prevalence of obese children in China is still lower than that in western countries, research on Chinese and other Asian immigrants in the US has shown that the longer the time spent in the US, the greater the risk of obesity [4], suggesting a significant environmental impact on the development of obesity. Thus, with the rapid economic development in China, and without intervention to prevent it, obesity in children and adults may become a major public health problem in this country.

Obesity has well documented adverse physical health consequences both in childhood and adulthood, including increased risk of cardiometabolic disease and some cancers [5–8]. In addition there is growing evidence that obesity in childhood and adolescence has a detrimental effect on health-related quality of life (HRQOL) [9–14], with severely obese children having HRQOL that is comparable to that of children with cancer [15]. HRQOL is a comprehensive and multi-dimensional construct that includes self-assessment of physical, emotional and social well-being [16]. Dimensions of HRQOL that appear to be most strongly associated with obesity are physical, social and school functioning [9, 17–19]. However, there is evidence to suggest that the impact of childhood obesity on HRQOL is influenced by culture [20], and may differ between boys and girls and with age, from childhood to adolescence [14, 21]. In some communities obesity is not recognized as a problem and is associated with good health, so one would expect HRQOL to be less influenced by obesity in these communities [22–24]. In addition to the relationship between obesity and HRQOL, the potential effect of underweight on HRQOL should also be considered, although some population studies have reported that the HRQOL of underweight youth was generally no different from those with normal weight [25]. To date, most studies that examine the relationship between weight status and HRQOL have been conducted in western or other high income countries and much of the evidence for an adverse relationship is based on studies in clinical populations [9, 17], the effect of weight status on HRQOL among children living in the community in lower income countries, such as China, has been rarely studied.

The aim of this study was to evaluate the association between HRQOL and weight status in a community sample of school children aged 8–12 years in Guangzhou, China.

## Methods

### Sampling and participants

The analysis presented comes from a sub-group of participants drawn from a larger study; a cross-sectional study undertaken in Guangzhou, which aimed to determine the prevalence and risk factors for overweight and obesity in children age 5–12 years. To achieve a representative sample, a multi-stage stratified cluster random sampling method was used. First, five of the ten urban districts in Guangzhou were selected using a random number generator. Second, within each selected district, schools were stratified by public or private status. Children who are permanently resident in Guangzhou are eligible to attend public schools, whereas children of economic migrant families are obliged to attend private schools. Six primary schools were randomly chosen from each district with a 2:1 (public: private school) ratio, therefore 4 public and 2 private schools were selected. Third, within each school two classes per grade (from grade 1 to 5) were randomly selected. Finally, all pupils from selected classes (age 5–12 years) were invited to take part. Children were excluded if they had a significant physical and psychological condition that was felt by teaching staff to compromise their participation in the study (e.g. children with a major disability, or serious cognitive or psychological dysfunction). Of the 30 selected schools, permission for the study was not obtained for one school (a private school), leaving 29 participating schools.

Written informed consent was sought from the parents of 11445 eligible children aged 5–12 years (on behalf of their children), 9917 (86.6%) of which agreed to participate. Data collection took place from April to June 2014. All participating children had anthropometric measurements taken in school by trained research staff using standardized procedures and equipment. The parents of all participating children were asked to complete a questionnaire which included questions about sociodemographic and lifestyle characteristics. Given the inherent difficulties of collecting survey data from young children [26], we administered a self-completion student questionnaire, which included a measure of HRQOL to children in grade 3 and above only (8+ years; *n* = 5962). After exclusion of 181 student questionnaires which were incomplete, the number of children aged 8–12 years included in this analysis was 5781.

The study was approved by the Ethical Committee of Guangzhou Center for Disease Control and Prevention and the University of Birmingham Ethics Committee. Permission to conduct the study in the identified schools was granted by the relevant Departments of Education and Health.

### Measurement of weight status

Children wore light clothing with no shoes for height and weight measurements. Height was measured to the nearest 0.1 cm using a TGZ type height tester (Dalian). Weight was measured to the nearest 0.1 kg using an electronic scale (JH-1993 T, weighing Apparatus Co. Ltd. Dalian). Body Mass Index was calculated (weight (kg)/Height (m)^2^) and standard deviation score (BMI z-score) derived using the age and sex specific WHO reference 2007 for children aged 5–19 years. Children were also categorized as underweight (<−2SD), healthy weight (between -2SD and 1SD), overweight (>1SD) and obese (>2SD) [27]. Measuring devices were systematically calibrated.

### Measurement of HRQOL

The PedsQL 4.0 was used to assess HRQOL in this study. This is a validated 23-item questionnaire for children aged 2–18 years [28], administered as either a child self-report or a parent proxy-report. Detailed information about the questionnaire has been reported elsewhere [28]. In brief, the PedsQL comprises four subscales: physical (8 items), social (5 items), emotional (5 items), and school functioning (5 items). Mean scores are calculated based on a 5-point response scale for each item and transformed to a 0–100 scale with a higher score representing better quality of life. The PedsQL subscales can be used to derive three summary scores: a total score (mean of all items), a physical health score (mean of physical functioning items) and a psychosocial health score (mean of emotional, social and school functioning items).

### Collection of parental data

Parents were asked to complete a questionnaire which included questions on level of maternal and paternal education, maternal and paternal height and weight (to calculate BMI), and family income.

### Statistical analyses

All statistical analyses were performed using SPSS for windows (version 21.0, SPSS, Inc., Chicago, IL). Descriptive statistics were used to summarise demographic anthropometric and HRQOL data. To explore differences in HRQOL between different subgroups independent-sample t tests (two groups) or general linear models (three groups) were used.

To explore the association between weight status and HRQOL, taking into account the effect of school as cluster, multilevel random effects models were developed with HRQOL scores (total, physical summary, psychosocial summary, emotional functioning, social functioning and school functioning scores) as dependent, and weight status (grouped into underweight, healthy weight (reference group), or overweight/obese) as the independent variables. Other covariates (gender, age group, school type, maternal education, paternal education and family income) were included in the multilevel models if statistically significant differences in HRQOL were found between the subgroups in the univariate analyses. School was added as a random effect and was significant in all models (*p* < 0.01). In addition, we conducted subgroup analyses to explore differences in the association between weight status and HRQOL in boys and girls, in younger (8–9 years old) and older (10–12 years old) age groups, and by type of school (public or private) attended. Level of significance in the analyses was set at 0.05.

## Results

### Participant characteristics

The characteristics of participating children are shown in Table 1. The average age was 9.7 years (SD = 1.01), and 54.5% were boys. Approximately 70% were public school students. The mean BMI z-score was −0.1 (SD = 1.33, range −4.07 to 5.47), although more than 20% of the children were overweight (13.0%) or obese (7.4%). The prevalence of overweight/obesity was higher in the public compared with the private schools (22.4% and 15.9% respectively, *χ* ^2^ = 31.79, *p* < 0.01).

**Table 1:** Comparison of unadjusted mean health related quality of life (HRQOL) total score (measured with PedsQL) among children with different demographic and socioeconomic characteristics

	n (%)	Unadjusted mean (s.d.) HRQOL total score	*P*
Total	5781 (100)	78.88 (13.57)	
Gender			
Male	3151 (54.51)	78.42 (13.87)	0.005
Female	2630 (45.49)	79.43 (13.17)	
Age group			
< 10	2536 (43.87)	77.93 (14.06)	<0.001
> =10	3245 (56.13)	79.63 (13.12)	
School type			
Private school	1743 (30.15)	75.36 (13.76)	<0.001
Public school	4038 (69.85)	80.40 (13.19)	
Children weight category			
Underweight	375 (6.49)	77.82 (13.10)	
Healthy weight	4226 (73.10)	78.94 (13.68)	0.288
Overweight/obesity	1180 (20.41)	79.02 (13.30)	
Maternal weight category (*n* = 5075)			
Healthy weight	4095 (80.69)	79.23 (13.24)	0.114
Overweight/obesity	980 (19.31)	78.44 (14.16)	
Paternal weight category (*n* = 4974)			
Healthy weight	3001 (60.33)	79.1813.38)	0.603
Overweight/obesity	1973 (39.67)	78.98 (13.50)	
Maternal education years (*n* = 5460)			
< =9	2801 (51.30)	76.80 (13.63)	
10–12	1355 (24.82)	80.40 (12.69)	<0.001
> 12	1304 (23.88)	82.25 (12.75)	
Paternal education years (*n* = 5451)			
< =9	2375 (43.57)	76.92 (13.66)	
10–12	1636 (30.01)	79.42 (13.16)	<0.001
> 12	1440 (26.42)	81.91 (12.74)	
Family income (*n* = 5075)			
Low	1941 (38.25)	77.25 (13.82)	
Medium	2088 (41.14)	79.43 (13.16)	<0.001
High	1046 (20.61)	81.51 (12.72)	

As shown in Table 1, participants reported an overall mean total HRQOL score of 78.88 (SD = 13.57). The unadjusted mean total HRQOL score in girls was slightly higher than in boys (*t* = 2.83, *p* = 0.005), and older children (10–12 years old) had higher HRQOL score than younger children (8–9 years old) (79.63 and 77.93 respectively, *t* = 4.70, *p* < 0.001). The HRQOL score of public school students was much higher than private school students (80.40 and 75.36 respectively, *t* = 13.14, *p* < 0.001). There was also a statistically significant trend for higher HRQOL scores with increasing years of parental education and family income. However, there was no significant difference in total HRQOL score across the three weight categories (*F* = 1.25, *p* = 0.288).

### Weight status and HRQOL

Table 2 shows the results of the adjusted models (after adjustment for gender, age, school type, length of maternal education, length of paternal education, family income). HRQOL scores across all domains were generally lower in both overweight/obese and underweight children compared with those who were healthy weight, although the differences were only statistically significantly lower for social functioning in the overweight/obese group (*β* = −1.93, *p* = 0.001) and total (*β* = −1.47, *p* = 0.05) and physical summary scores (*β* = −2.18, *p* = 0.02) among underweight children, compared with those who were healthy weight.

**Table 2:** Comparison of PedsQL scores among different weight status groups after adjustment for covariates^a^

	Adjusted mean (SE)			Underweight vs. Healthy weight			Overweight/obesity vs. Healthy weight		
	Healthy weight	Underweight	Overweight/obesity	*β*	*SE*	*P*	*β*	*SE*	*P*
Total PedsQL score	79.0 (0.5)	77.5 (0.8)	78.1 (0.6)	−1.47	0.75	0.05	−0.84	0.48	0.08
Physical summary score	80.3 (0.6)	78.1 (1.0)	79.5 (0.7)	−2.18	0.90	0.02	−0.81	0.57	0.15
Psychosocial summary score	78.3 (0.5)	77.2 (0.9)	77.5 (0.7)	−1.10	0.81	0.17	−0.84	0.51	0.10
Emotional functioning score	72.1 (0.7)	71.7 (1.3)	72.1 (0.9)	−0.45	1.14	0.69	−0.04	0.72	0.95
Social functioning score	85.9 (0.5)	84.4 (1.0)	84.0 (0.7)	−1.48	0.93	0.11	−1.93	0.59	0.001
School functioning score	76. 8 (0.6)	75.5 (1.1)	76.3 (0.8)	−1.39	0.96	0.15	−0.50	0.60	0.41

^a^Adjusted for gender, age, school type, maternal education years, paternal education years, family income

### Weight status and HRQOL in subgroups

Subgroup analysis by gender, school type and age group are shown in Tables 3, 4 and 5 respectively. For boys, there was no significant association between overweight/obesity and HRQOL in any dimension of PedsQL. However, underweight boys had lower social functioning scores (*β* = −3.63, *p* = 0.01). In girls, the overweight/obese group had significantly lower total (*β* = −1.96, *p* = 0.02), psychosocial (*β* = −2.40, *p* = 0.01), social functioning (*β* = −3.36, *p* = 0.001), and school functioning (*β* = −2.19, *p* = 0.03) scores than the healthy weight group. Physical summary scores were significantly lower in underweight compared with healthy weight girls (*β* = −2.27, *p* = 0.047). In addition, we found underweight boys had significantly lower social functioning scores than underweight girls (*t* = 5.5, *p* < 0.01).

**Table 3:** Comparisons of PedsQL scores among children with different weight status after adjustment for covariates^a^, stratified by gender

		Adjusted mean (SE)			Underweight vs. Healthy weight			Overweight/obesity vs. Healthy weight		
		Healthy weight	Underweight	Overweight/obesity	*β*	*SE*	*P*	*β*	*SE*	*P*
Boys	Total PedsQL score	78.3 (0.6)	76.4 (1.3)	78.0 (0.7)	−1.97	1.16	0.09	−0.30	0.60	0.61
	Physical summary score	80.9 (0.8)	78.6 (1.5)	80.2 (0.9)	−2.31	1.40	0.10	−0.70	0.72	0.33
	Psychosocial summary score	76.9 (0.6)	75.1 (1.3)	76.8 (0.8)	−1.78	1.25	0.15	0.07	0.64	0.91
	Emotional functioning score	71.7 (0.9)	71.6 (1.8)	72.6 (1.1)	−0.15	1.70	0.93	0.90	0.87	0.30
	Social functioning score	84.4 (0.7)	80.8 (1.5)	83.2 (0.8)	−3.63	1.47	0.01	−1.28	0.76	0.09
	School functioning score	74.5 (0.7)	73.0 (1.6)	74.8 (0.9)	−1.53	1.51	0.31	0.25	0.77	0.74
Girls	Total PedsQL score	79.8 (0.6)	78.5 (1.1)	77.8 (0.9)	−1.33	0.98	0.18	−1.96	0.81	0.02
	Physical summary score	79.7 (0.7)	77.5 (1.3)	78.6 (1.1)	−2.27	1.15	0.047	−1.14	0.94	0.23
	Psychosocial summary score	79.8 (0.7)	79.0 (1.2)	77.4 (1.0)	−0.83	1.06	0.44	−2.40	0.87	0.01
	Emotional functioning score	72.8 (0.9)	71.7 (1.7)	71.1 (1.4)	−1.09	1.55	0.48	−1.66	1.27	0.19
	Social functioning score	87.5 (0.7)	87.5 (1.3)	84.1 (1.1)	0.04	1.18	0.97	−3.36	0.97	0.001
	School functioning score	79.3 (0.8)	77.8 (1.4)	77.1 (1.2)	−1.48	1.22	0.22	−2.19	1.00	0.03

^a^Adjusted for age, school type, maternal education years, paternal education years, family income

**Table 4:** Comparisons of PedsQL scores among children with different weight status after adjustment for covariates^a^, stratified by school type

		Adjusted mean (SE)			Underweight vs. Healthy weight			Overweight/obesity vs. Healthy weight		
		Healthy weight	Underweight	Overweight/obesity	*β*	*SE*	*P*	*β*	*SE*	*P*
Public school	Total PedsQL score	81.2 (0.7)	79.8 (1.0)	80.5 (0.8)	−1.39	0.87	0.11	−0.69	0.54	0.20
	Physical summary score	83.2 (0.7)	80.8 (1.2)	82.4 (0.9)	−2.38	1.02	0.02	−0.72	0.63	0.25
	Psychosocial summary score	80.1 (0.7)	79.2 (1.1)	79.4 (0.8)	−0.84	0.95	0.37	0.64	0.58	0.27
	Emotional functioning score	74.8 (1.0)	74.9 (1.6)	75.1 (1.2)	0.10	1.34	0.94	0.29	0.83	0.73
	Social functioning score	86.6 (0.7)	85.6 (1.2)	85.0 (0.8)	−0.99	1.07	0.35	−1.65	0.66	0.013
	School functioning score	78.8 (0.8)	77.2 (1.3)	78.3 (1.0)	−1.63	1.11	0.14	−0.49	0.69	0.48
Private school	Total PedsQL score	77.0 (0.7)	75.2 (1.6)	75.6 (1.1)	−1.82	1.49	0.22	−1.46	1.01	0.15
	Physical summary score	77.1 (1.1)	75.3 (2.1)	75.8 (1.5)	−1.73	1.85	0.35	−1.31	1.25	0.29
	Psychosocial summary score	76.9 (0.7)	75.0 (1.6)	75.3 (1.1)	−1.91	1.56	0.22	−1.54	1.05	0.14
	Emotional functioning score	69.9 (1.0)	67.6 (2.3)	68.6 (1.5)	−2.24	2.16	0.30	−1.30	1.46	0.37
	Social functioning score	85.0 (1.1)	82.4 (2.1)	82.3 (1.5)	−2.64	1.88	0.16	−2.77	1.27	0.029
	School functioning score	75.1 (0.9)	73.8 (2.0)	74.5 (1.4)	−0.78	1.87	0.68	−0.64	1.26	0.61

^a^Adjusted for gender, age, maternal education years, paternal education years, family income

**Table 5:** Comparisons of PedsQL scores among children with different weight status after adjustment for covariates^a^, stratified by age

		Adjusted mean (SE)			Underweight vs. Healthy weight			Overweight/obesity vs. Healthy weight		
		Healthy weight	Underweight	Overweight/obesity	*β*	*SE*	*P*	*β*	*SE*	*P*
Age < 10	Total PedsQL score	77.9 (0.6)	76.4 (1.3)	77.3 (0.8)	−1.56	1.25	0.21	−0.64	0.77	0.41
	Physical summary score	79.3 (0.7)	78.1 (1.5)	80.1 (1.0)	−1.27	1.49	0.40	0.79	0.92	0.39
	Psychosocial summary score	77.2 (0.6)	75.4 (1.4)	75.8 (0.9)	−1.75	1.34	0.19	−1.37	0.83	0.10
	Emotional functioning score	71.6 (0.8)	69.7 (1.9)	70.6 (1.2)	−1.90	1.87	0.31	−0.99	1.15	0.39
	Social functioning score	84.2 (0.6)	82.8 (1.6)	82.4 (1.0)	−1.41	1.56	0.36	−1.82	0.96	0.06
	School functioning score	75.8 (0.7)	73.7 (1.6)	74.6 (1.0)	−2.04	1.57	0.20	−1.19	0.97	0.22
Age > =10	Total PedsQL score	80.0 (0.5)	78.5 (1.0)	79.1 (0.7)	−1.51	0.94	0.11	−0.94	0.60	0.12
	Physical summary score	81.4 (0.6)	78.5 (1.2)	79.5 (0.8)	−2.90	1.10	0.009	−1.87	0.71	0.009
	Psychosocial summary score	79.3 (0.5)	78.5 (1.0)	78.9 (0.7)	−0.75	1.01	0.46	−0.43	0.65	0.51
	Emotional functioning score	73.0 (0.7)	73.5 (1.5)	73.7 (1.0)	0.47	1.43	0.74	0.72	0.92	0.43
	Social functioning score	86.9 (0.5)	85.3 (1.2)	85.0 (0.7)	−1.64	1.16	0.16	−1.91	0.74	0.01
	School functioning score	78.0 (0.6)	76.9 (1.2)	77.0.8)	−1.07	1.20	0.37	−0.06	0.77	0.94

^a^Adjusted for gender, school type, maternal education years, paternal education years, family income

Regarding school type, children from public schools had higher scores in all dimensions of PedsQL than children from private schools across all weight categories, but the relationship between HRQOL and overweight/obesity did not differ by school type. Inverse association was just founded between overweight/obesity and social functioning in both public and private schools (*β* = −1.65, *p* = 0.013; and *β* = −2.77, *p* = 0.029 respectively). Significantly lower physical summary scores in the underweight compared to the healthy weight group was only seen in public schools (*β* = −2.38, *p* = 0.02).

There were no significant associations between weight status and HRQOL in children under 10 years, but in those aged 10 and over, physical summary scores were lower in both the overweight/obese group and the underweight group compared with the healthy weight group (*β* = −1.87, *p* = 0.009; and *β* = −2.90, *p* = 0.009 respectively). Social functioning scores were lower in overweight/obese children compared with healthy weight children only in this older age group (*β* = −1.91, *p* = 0.01).

## Discussion

Overall, the study findings did not provide evidence for a strong association between overweight/obesity and health related quality of life among children aged 8 to 12 years in Guangzhou, China. Although our study indicated that overweight/obese children reported significantly lower scores in social functioning than healthy weight children, the absolute difference in scores was small, particularly in relation to the larger effect of social factors (such as parental education, income and migrant status) on HRQOL. Our findings in relation to weight status are in keeping with other studies conducted in low income countries. Studies in Fiji (with 8947 children aged 12–18 years) [29], and Kuwait (with 500 children aged 10–14 years) [30], both reported that HRQOL was similar in obese and healthy weight children. Another smaller study with 778 children, aged 6–13 years in Guangzhou in China also showed that, except for school functioning, obesity had no significant association with HRQOL [31]. A further study with 98 pairs of obese adolescents and healthy weight peers in Kuwait found that only the physical dimension of HRQOL was lower in the obese participants [30]. However, the findings of this study contrast with several other studies which suggest that overweight and obesity could significantly impair children’s HRQOL [9–12, 17, 21, 32]. There may be two potential reasons for this. Firstly, much of the evidence for an adverse relationship was based on studies in clinical populations [9, 10, 12, 17, 32], which may have resulted in an overestimation of the strength of the association [14]. Cultural differences may be another factor. Similar to Fiji [29], most Chinese people, especially the elderly, expect children to be heavy, as this is taken as a sign of health and prosperity [26, 33]. Additionally, in this study we found that the total and physical summary scores were lower in the underweight group, particularly among older children. This contrasts with several studies which report no significant difference in HRQOL between underweight and healthy weight children [25, 34, 35]. Again, this could be linked to cultural influences; if heaviness is taken to be a sign of health, growth and prosperity, then underweight may indicate poor health and poverty, and thus lead to a lower quality of life.

Subgroup analyses revealed a different pattern of associations between weight and dimensions of HRQOL in certain groups. There were gender differences, with more prominent associations between psychosocial summary scores and overweight/obesity in girls, but not in boys. These findings are somewhat consistent with a Lebanese study conducted with adolescents, which found that obesity was related to all dimensions in girls except emotional and school functioning, but in boys, only the inverse association between obesity and physical functioning was significant [36]. Other studies have also reported associations between obesity and lower HRQOL scores in girls, but not in boys [14, 37]. A potential explanation for this may be the difference between boys and girls in the perception of ideal body image. Influenced by television, magazines, advertisements and Chinese culture, thinness represents the ideal body image for girls, but not for boys. A previous study (with 12750 participants aged 10–26 years) conducted in Guangzhou showed that 47.18% girls with healthy weight perceived themselves as being overweight, but this percentage was just 18.08% in boys. Furthermore, 28.55% healthy weight boys expected to be bigger [38]. This different perception between genders of the ideal body shape is coherent with the finding of lower psychosocial quality of life in girls who are overweight and lower social functioning scores in boys who are underweight. A French study, reported similar findings, with higher BMI in girls and lower BMI in boys associated with lower mental HRQOL scores [39]. We also found that the association between overweight/obesity and HRQOL was more apparent in the older age group (10–12 years) affecting both physical and social functioning. A potential explanation for the impact of weight status on physical functioning as children get older, is that the physical manifestations of both overweight and underweight become more prominent with increasing age. This is in line with a study by Fontaine, in which the authors reported that the impact of obesity on physical functioning was significantly more than its impact on psychological functioning in adults [40].

Although the association between weight status and HRQOL was similar across the different school types, children in public schools had higher scores in almost all domains of HRQOL compared with those in private schools. A possible explanation for this is that most of the children attending private schools are migrants. Migrant families tend to be less affluent and less well educated relative to the resident families whose children attend the public schools [41].

In contrast to previous studies, we found that HRQOL scores overall were higher in older children. Most previous research reports lower HRQOL in adolescents compared with children [42–44], although the majority of these studies were undertaken in western countries, and may reflect cultural differences, such as differences in parental and peer relationships and in adolescent expectations.

A strength of this study is that it is one of the first and larger studies to explore the relationship between childhood weight status and HRQOL in a country undergoing rapid economic transition. Also, the findings will be generalizable to other Chinese urban populations as they are drawn from a large representative sample of both resident and migrant communities. The study also has some limitations. We only measured HRQOL from the child’s perspective. Parent proxy-reported HRQOL would have enabled triangulation of findings and potentially more robust conclusions could have been drawn. A further limitation is the cross-sectional nature of the study, which means a temporal relationship between weight status and quality of life cannot be explored.

## Conclusions

This study has demonstrated that the relationship between overweight/obesity and HRQOL in children in China is not as prominent as that seen in children in western or high-income countries. However, the gender differences in the association between adiposity and HRQOL that exist in other populations have been observed to a certain extent in this study. In Chinese populations, underweight may be an important contributor to lower HRQOL. Future intervention to prevent both over and undernutrition may improve quality of life in both the underweight and the overweight and obese groups.

## Abbreviations

BMI: Body mass index
HRQOL: Health-related quality of life
SD: Standard deviation
WHO: World Health Organization
